# The outcome of medical nutrition therapy on glycemic control among type 2 diabetic patients

**DOI:** 10.11604/pamj.2023.45.85.35520

**Published:** 2023-06-15

**Authors:** Shimaa Elsayed Abdelsalam, Mosleh Abdel Rahman Ismaail, Eman Ahmed Sultan, Omneya Ezzat Elsherif, Hend Mikhail Salama, Shimaa Ibrahim Hassan

**Affiliations:** 1 Department of Family Medicine, Faculty of Medicine, Suez Canal University, Ismaïlia, Egypt,; 2 Department of Endocrinology, National Institute of Nutrition, El Sayeda Zeinab, Egypt,; 3 Cairo University Hospitals, Cairo University, Cairo, Egypt

**Keywords:** Glycemic control, medical nutrition therapy, type 2 diabetes

## Abstract

**Introduction:**

the global prevalence of type 2 diabetes in adults is estimated to be 6.4%. The current prevalence of type 2 diabetes in Egyptians aged 20 to 79 is approximately 15.6%. The objective of medical nutrition therapy (MNT) is to optimize the management of the “ABC” for diabetes control, glycated hemoglobin, blood pressure, and cholesterol. Our study aimed to assess the effect of MNT on glycemic control in patients with type 2 diabetes attending the family practice clinic.

**Methods:**

a quasi-experimental intervention trial was conducted with 40 diabetic patients seeking medical service in the Suez Canal University Hospital family practice clinic. The participants were over 20 years old and had uncontrolled type 2 diabetes. Patients were surveyed using El-Gilany questionnaire to evaluate the socio-demographic traits, physical examination data, and laboratory investigations at baseline and after 12 weeks.

**Results:**

medical nutrition therapy (MNT) significantly reduced glycated hemoglobin level (p<0.001); the median level of glycated hemoglobin was 10% with a minimum level of 7.5% vs. a maximum of 14% in the pre-intervention phase. In comparison, the median glycated hemoglobin level was 9.5%, with a minimum level of 5.6% vs. a maximum of 13.5% in the post-intervention phase. In addition, there was a significant improvement in blood pressure, weight, body mass index, fasting plasma glucose, and lipid profile. **Conclusion:** there is evidence that MNT is a crucial component of type 2 diabetes therapy.

## Introduction

The global prevalence of diabetes will rise to 10.2% (578 million) by 2030 and 10.9% (700 million) by 2045 [1]. The number of adults with diabetes is expected to rise to 10.4% by 2040 [2]. The number of adults with diabetes in the Middle East and North Africa (MENA) Region is projected to reach 95 million by 2030 and 136 million by 2045. In 2021, diabetes-related mortality reached 796,000 in 2021 [3]. Approximately 15.6% of all persons in Egypt between the ages of 20 and 79 now have type 2 diabetes (T2DM) [4]. The rise in diabetes is negatively impacting Egypt's healthcare system. Diet is one of the essential behavioral components of diabetes management. Medically tailored meals may improve outcomes and reduce healthcare expenditures [5]. However, principles of nutritional management are not fundamentally understood by professionals or their patients. Nowadays, research has demonstrated the clinical efficacy of medical nutrition therapy (MNT) in diabetes based on empirical evidence. The American Dietetic Association (ADA) introduced the phrase “MNT” in 1994. It contains two phases: 1) assessment of the client's nutritional state and 2) treatment, which includes nutrition therapy, counseling, and using specialist nutritional supplements [6]. The MNT aims to control the “ABC” of diabetes control: glycated hemoglobin (HbA1c), blood pressure (BP), and low-density lipoprotein (LDL) cholesterol. The prescription must be modified for the individual patient to treat any present or potential complications from diabetes or other concomitant illnesses [7]. The MNT in our study involves the following: 1) an evaluation of the patient's knowledge and skills in diet and diabetes self-management; 2) individualized nutrition goals; 3) nutritional intervention that involves a careful match of both a meal-planning strategy and educational materials to the patient's needs, with freedom for the patient to execute; 4) evaluation of outcomes and ongoing monitoring. This study aimed to assess the outcome of MNT on glycemic control for T2DM patients attending the family practice (FP) clinic at Suez Canal University Hospital (SCUH). In addition, we aimed to improve glycemic control among T2DM patients through the MNT education program.

## Methods

**Study design and sampling method:** a quasi-experimental interventional study (pre and post-test design) was carried out on 40 diabetic patients by using the following equation [8]:


$$n=2{\left[\frac{\left(Z_{\infty /2}+Z_{\beta }\right)*\sigma }{\mu_{1}-\mu_{2}}\right]}^{2}$$

Where n is sample size, µ_1_= mean of HbA1c level of diabetic patients before the intervention of health education =10.41 [9] and µ_2_= mean of HbA1c level of diabetic patients after the intervention of health education =8.22 [9]. Based on the above equation, the calculated sample was 12 participants. Considering the 20% dropout, the total sample size was 15 participants, and we extended the study sample size to 40. The study was conducted in the family practice (FP) clinic at SCUH, the principal university teaching hospital in the Ismailia governorate. In 2021, a non-probability convenient sampling strategy was used to acquire the sample units for uncontrolled T2DM patients, attending the FP clinic seeking medical care. The practical component of the study was fulfilled by attending the clinic for 15-20 minutes twice a month over 3 months period.

### Study participants and data collection

**Inclusion criteria:** the inclusion criteria were T2DM patients > 20 years of age, capable of providing written informed consent, and with uncontrolled diabetes (HbA1c >7%), presenting to the FP clinic [10].

**Exclusion criteria:** the exclusion criteria were pregnant women (to avoid ketoacidosis due to low calories) [10], patients with recent cardiovascular complications (e.g., myocardial infarction, stroke, and congestive heart failure), and patients who are currently involved in a similar nutritional educational program (to avoid contamination bias).

In the first part of the study, the El-Gilany *et al*. 2012 [11] questionnaire was used to measure the patient's socio-demographic characteristics encompassing seven categories: (education, culture, occupation, family, home cleanliness, economic, and healthcare domains) with a total score of 84. The second component evaluated the patient's medical history, including length of diabetes, therapeutic regimen, other medical therapy, concomitant conditions, and medication adherence. In the present study, we utilized the four-item Morisky medication adherence scale (MMAS), which consists of four yes/no questions. The MMAS yields a score between 0 and 4, and the developers proposed three levels of medication adherence based on this score: high, medium, and low adherence with 0, 1-2, and 3-4 points, respectively [12]. In the third part, we evaluated physical examination data, such as anthropometric measurements (weight, height, BMI) and blood pressure (BP). The fourth part was the assessment of the laboratory investigations: HbA1c, fasting plasma glucose (FPG), and lipid profile [13]. The fifth part was about performing dietary assessment; twenty-four-hour dietary recall forms were used for nutritional evaluation of the studied sample according to the American Nutrition Association (ANA) 2014 [14]. This provided information about the patient's current diet, which enabled the researchers to build up an appropriate nutritional educational program for the group, with individualized tailoring. Correspondingly, this nutritional assessment was used to help compare the dietary habits of the studied group (at zero time and every 2 weeks).

Nutrition assessment, nutrition diagnosis, nutrition intervention, and nutrition monitoring and evaluation are the four standard steps of the MNT program for diabetic patients. These steps can be summarized as follows:

**Nutrition assessment:** evaluating a person's nutrition status is an essential initial step in commencing nutritional care. The component of the nutritional evaluation is data acquisition which includes: A) Anthropometric data (weight, height, and BMI). B) Biochemical data (HbA1c, fasting or non-fasting plasma glucose, and lipid profile). C) Clinical data (signs and symptoms of the disease(s), problems relating to intake (such as chewing, swallowing, gastrointestinal problems), blood pressure diagnosis, and treatment information). D) Dietary data (diet history as an individual to record or describe what, how much, and when he or she typically consumes food within a 24-hour period may be the most valuable).

**Nutrition diagnosis:** it is described in the PES format, which includes the problem (P), the cause (E), and the signs and symptoms (S). For example, some patients are diagnosed as having inappropriate fat consumption(P) associated with inadequate knowledge (E), as shown by a high intake of foods containing saturated fats (S).

**Nutrition intervention:** it consists of two steps; setting up a nutrition care plan based on a well-balanced diet and implementing that plan.

**Nutrition monitoring and evaluation:** plans for the next appointment should be established before the patient departs the session. A timetable for follow-up visits should be created to monitor and evaluate the efficacy of dietary changes. In order to plan for the next appointment, the patient is instructed to maintain a 3-day or weekly meal log that includes blood glucose monitoring data.

There were 3 stages of applying the previous four steps.

**Pre-intervention:** a questionnaire that lasted for almost 20 minutes was filled out. Basal assessment of weight, height, BMI, BP, FPG, and HbA1c, and in addition, dietary assessment were recorded.

**Intervention stage:** the MNT intervention 12-week program included 6 sessions held in the FP outpatient clinic. The first session lasted for 60 minutes. It started with an introduction to the program and the initiation of the first diabetes MNT. The subsequent 5 sessions were 15-20 minutes for follow-up and reinforcement and were performed individually (face-to-face or phone call). These follow-up sessions are intended to encourage diabetic patients to adhere to the nutritional intervention, adopt the structured food plan, and address any concerns that may arise during the intervention. Every patient received a copy of the food intake record form. Based on their baseline evaluation, the participants were given teaching materials illustrating the “plate method” and healthy eating tips. The main idea was to educate the patients on properly planning their meals; half the meal is composed of non-starchy vegetables, and the rest is equally distributed between lean proteins, starchy vegetables, and whole grain carbohydrates. Participants also got instructional materials to help them make healthier choices within each food group and exchange list, as well as instructions to follow a hypocaloric dietary plan (1500 kcal/day for women, 1800 kcal/day for men) to improve HbA1c and reduce body weight by 5% to 10%.

**Post-intervention:** this stage started two weeks after completion of the last session (weight, height, BMI, BP, FPG, HbA1c, lipid profile, and dietary assessment were evaluated).

**Outcome measures:** after 12 weeks of intervention, the primary endpoint of this study was the impact of MNT on HbA1c. The secondary outcomes were the change in body weight, BP, lipid profile, and FPG.

**Administrative approval:** the study was approved by Suez Canal University, Faculty of Medicine (SCU). The head of the Family Medicine Department at SCU, Faculty of Medicine, provided official approval.

**Ethical approval:** ethical considerations for the study included giving participants a written consent form that explained the purpose and the nature of the study. We used coding by numbers for the study participants to guarantee confidentiality. The Institutional Review Board (IRB) granted us formal approval number 3903 (July 2019).

**Data management:** the statistical package for social sciences (SPSS) software, version 24, was used to analyze the data. Continuous quantitative data, such as age, were expressed as mean, standard deviation (SD), median, and interquartile range (IQR), while categorical qualitative variables were expressed as absolute and relative frequencies (%). After establishing normality with the Shapiro-Wilk test, the required tests of statistical significance were conducted. The Wilcoxon signed-rank test was used to compare identical results. The results were judged statistically highly significant when the significance probability was less than 0.05.

## Results

**Figure 1:**
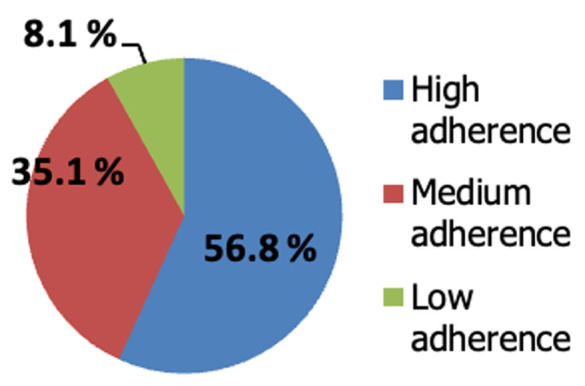
frequency distribution of studied diabetic patients regarding their levels of adherence according to MMAS

The current study recruited 40 diabetic patients aged 35 to 64 years old. Thirty-two (80%) participants were females, while 8 (20%) were males. Moreover, 87.5% of men were employed, while just 25% of women were, and 50% of women were illiterate compared to 17.5% of men. Table 1 shows that more than half of the studied patients were residents of Urban/Urban slum areas (65%), with only 5.4 % belonging to a higher social class. In addition, more than half of the participants (57.5%) can just meet their routine daily expenses. Table 2 reveals that over 55% had diabetes for over ten years. Combinations of metformin and insulin (50 percent) and metformin plus sulfonylurea (37.5%) were the most frequently prescribed drugs. In addition, around 72.5% of patients have additional comorbid conditions such as hypertension (35%) and dyslipidemia (47.5%). Figure 1 shows that 56.8% of the patients have a high level of adherence, while 8.1% of the patients have a low level of adherence to medications, respectively, according to MMAS. The figure demonstrates that though the studied diabetic patients were highly adherent to medications, they were less adherent to the diet. The implementation of the MNT program resulted in higher adherence to the diet. Table 3 shows the clinical outcome, where the median BMI at baseline was 33.6 kg/m^2^ and ranged from 26 to 48 kg/m^2^, while the median BMI post-intervention was 32.8 kg/m^2^ with a range from 70 to 90 kg/m^2^, with a mean reduction of 1.10 ± 0.99 kg/m^2^, which was statically significant. Furthermore, systolic and diastolic blood pressure decreased after intervention by a mean of 7.97 ± 9.82 and 4.86 ± 6.29 mmHg, respectively. Table 4 shows another laboratory change, as FPG median level in the pre-intervention was 215 mg/dl and ranged from 150 to 492 mg/dl, while the median level after the intervention was 150 mg/dl and ranged from 110-280 mg/dl, which is statistically significant (P < 0 .001). Moreover, a significant reduction in the lipid profile was observed with the intervention (P < 0.001). At baseline, study participants had a mean±SD HbA1c of 10.30 ± 1.83% ranging from 7.5 to 14%. After 12 weeks, the mean HbA1c was 9.72 ± 1.82%, ranging from 5.6 to 13.5%. A significant reduction in HbA1c (0.68 ± 0.40%) was observed following MNT (P < 0.001). Table 5 shows that there is a significant difference between socioeconomic status (SES) groups and HbA1c (p=0.027). Patients with high SES had considerably greater HbA1c than those with low or very low SES (p=0.048). Moreover, a strong positive association exists between BMI value and intervention (r= 0.778, P <0.001).

**Table 1:** frequency distribution of the studied diabetic patients according to their socioeconomic characteristics (N=40)

Socioeconomic Variables	Frequency	Percent (%)
**Age (years)**		
Mean ±SD	52.00 ± 7.69	
Median (range)	50(35-64)	
**Gender**		
Male	8	20%
Female	32	80%
**Residency**		
Urban/ Urban slum	26	65%
Rural	14	35%
**Husband's Education**		
Illiterate	7	17.5%
Read and write	11	27.5%
Primary school	2	5%
Preparatory school	5	12.5%
Secondary	8	20%
Intermediate	3	7.5%
University/ Postgraduate degree	4	10%
**Wife's Education**		
Illiterate	20	50%
Primary school	5	12.5%
Preparatory school	4	10%
Secondary	4	10%
Intermediate	2	5%
University/ Postgraduate degree	5	12.5%
**Husband's Occupation**		
Unemployed	5	12.5%
Unskilled manual worker	15	37.5%
Skilled manual worker/ Farmer	8	20%
Trades/business	4	10%
Semiprofessional/clerk	1	2.5%
Professional	7	17.5%
**Wife's Occupation**		
Housewife	30	75%
Unskilled manual worker	4	10%
Semiprofessional/clerk	1	2.5%
Professional	5	12.5%
**Crowding Index**		
≤ 1 person per room	14	35%
> 1 person per room	26	65%
**Income from all resources**		
In debt (inadequate)	10	25%
Just meet routine expenses (adequate)	23	57.5%
Meet routine expenses and emergencies (adequate)	6	15%
Able to save/invest money	1	2.5%

**Table 2:** frequency distribution of the studied diabetic patients according to their medical characteristics

Medical Variables	Frequency	Percent (%)
**Duration of diabetes mellitus**		
< 5 years	7	17.5%
5 -10 years	11	27.5%
> 10 years	22	55%
**Type of antidiabetic drugs**		
Metformin + Insulin	20	50%
Metformin + Sulfonylurea	15	37.5%
Metformin/ Sulfonylurea + DPP4	2	5%
Sulfonylurea only	2	5%
DPP4+ SGLT2	1	2.5%
**Comorbidities/complications**		
**Absent**	11	27.5%
**Present**	29	72.5%
Neurological disease	26	65%
Hypercholesterolemia	19	47.5%
Eye disease	16	40%
Hypertension	14	35%
Thyroid disease	1	2.5%

**Table 3:** pre-post clinical outcome changes among the studied diabetic patients

Clinical variables	Pre-intervention Mean ± SD Median (IQR)	Post-intervention Mean ± SD Median (IQR)	Mean difference	P-value
Systolic blood pressure mmHg	130.00 ± 13.64 130 (110-160)	122.02 ± 11.14 120 (110-150)	7.97 ± 9.82	< 0.001*
Diastolic blood pressure mmHg	82.43 ± 8.26 82 (70-95)	77.56 ± 6.83 80 (70-90)	4.86 ± 6.29	< 0.001*
Weight Kg	87.32 ± 11.01 85 (70-116)	84.30 ± 11.15 87 (67-114)	2.66 ± 2.56	< 0.001*
BMI Kg/m2	33.35 ± 4.59 33.6 (26-48)	32.20 ± 4.92 32.8 (70-90)	1.10 ± 0.99	< 0.001*

P-values are based on Wilcoxon signed-rank test. Statistical significance at P < 0.05.

**Table 4:** comparison of laboratory measures among the studied group before and after the intervention

Variables	Pre-intervention Mean ± SD Median (IQR)	Post-intervention Mean ± SD Median (IQR)	Mean difference	P-value
**Laboratory measures**				
Fasting plasma glucose mg/dl	238.81 ± 77.39 215 (150-492)	164.83 ±41.36 150 (110-280)	73.97 ± 51.1	< 0.001*
HbA1c (%)	10.30 ± 1.83 10 (7.5-14)	9.72 ± 1.82 9.5(5.6-13.5)	0.68 ± 0.40	< 0.001*
Total cholesterol (mg/dl)	203.75 ±56.72 209 (93-316)	176.77± 38.58 188 (100-250)	26.03 ± 37.13	< 0.001*
HDL cholesterol (mg/dl)	44.32 ± 9.78 45 (22-60)	46.12 ± 7.99 44 (32-60)	1.83 ± 4.11	0.023*
LDL cholesterol	131.64 ± 39.91 125 (60-216)	103.38 ± 28.93 108 (65-200)	28.54 ± 32.71	< 0.001*
Triglycerides	179.59 ± 82.37 170 (74-476)	150 ± 45.76 137 (100-250)	28.90 ± 59.97	0.005*

P-values are based on Wilcoxon signed-rank test. Statistical significance at P < 0.05.

**Table 5:** association of patients' socioeconomic and clinical characteristics with a change in HbA1c (N=40)

Variables	HbAlc (mean ± SD)	Test value	P-value
**Gender**			
Male	-0.76 ± 0.48	41	0.295a
Female	-0.43 ± 0.36		
**Socioeconomic level**			
Very low/ low	-0.37 ± 0.33	7.2	0.027*b
Middle	-0.68 ± 0.29		
High	-1.25 ± 0.35		
**Duration of DM, n (%)**			
< 5 years	-0.752 ± 0.32	1.16	0.446
5-10 years	-0.46 ± 0.50		
> 10 years	-0.44 ± 0.34		
**Comorbidities, n (%)**			
Absent	-0.65 ± 0.42	59	0.153a
Present	-0.41 ± 0.37		
**Morsiky Medication Adherence Scale level**			
Low	-0.30 ± 0.28	0.94	0.624
Medium	-0.40 ± 0.42		
High	-0.56 ± 0.39		

P-values are based on the Mann-Whitney U test. Statistical significance at P < 0.05. b p-values are based on the Kruskal-Wallis test. Statistical significance at P < 0.05.

## Discussion

This is an interventional trial to see how MNT affected how well T2DM patients controlled their blood sugar. The study involved 40 diabetic patients who attended FP outpatient clinics. HbA1c is the hallmark of glycemic control. The BMI decrease during the 12-week research period was 1.10 ± 0.99 kg/m^2^, and this result was the same in a study conducted in the USA [15] where BMI pre-intervention was 33.9 ±6.1 and the post-intervention mean reduction was 1.06 ± 0.5 kg/m^2^. However, this is not statistically significant (P = 0.13). Although this does not agree with a study conducted in Iran that showed that the mean BMI decreased by 0.5 ± 0.9 kg/m^2^ post-intervention, this difference was statistically significant (P = 0.022) [16]. This difference might be attributed to the study group's good adherence to the diet and the intervention program in the current study. A randomized controlled trial (RCT) was carried out in India to assess the effectiveness of MNT in managing T2DM, which showed a mean decrease of BMI of 0.5 kg/m^2^ from baseline after 3 months [17]. Regarding BP in the present study, the pre-intervention average systolic blood pressure (SBP) was 130.00 ± 13.64 mmHg, and the average diastolic blood pressure (DBP) was 82.43 ± 8.26 mmHg. In contrast, the post-intervention average SBP was 122.02 ± 11.14 mmHg, the average DBP was 77.56 ± 6.83 mmHg, and the mean difference in systole and diastole was 7.97 ± 9.82 and 4.86 ± 6.29, respectively, with a P value of < 0.001 for both. These findings are consistent with those in Iran, which reported a change in BP in systole and diastole mean differences of 5 ± 23 (P = 0.95) and 7 ± 9 (P= 0.06), respectively [16]. On the other hand, there was a discrepancy between this study and another study conducted in the USA, where no change in systolic or diastolic blood pressure occurred in the intervention group [15]. It could be due to the significant dropout rate in the previous study.

A 1 percent decrease in HbA1c is associated with a 37% decrease in the risk of microvascular complications and a 21% decrease in the risk of any endpoint or death attributable to diabetes [18]. HbA1c indicates a 0.68 ± 0.40 percent reduction in SD, as seen in the present study group. This illustrates the effectiveness of MNT in reducing T2DM in this population. A study in Alabama proved statistically significant HbA1c reductions of 0.9% to 1.9% [19]. This was also reported in a study conducted in 2006 among T2DM in Iran that showed HbA1c means SD decreased by 1.9 ± 2.1% in the intervention group. This more remarkable improvement in HbA1c compared to the current study could be explained by the different cultures of the studied patients and sample size [16]. In another RCT involving T2DM patients, Mexican Americans in Texas demonstrated a 1.5 percent reduction in the intervention group's 6-month HbA1c levels [20]. This agreed with a study in the USA which reported that the percentage of HbA1c decreased significantly in the intervention group (0.61%) (p < 0.001) [15]. This matching of the above and current studies could be attributed to using the same interventional program and participants' commitment. Similarly, a study in 2019 [7] in Iran found a significant reduction in HbA1c (0.43%). Another study in India reported that the group who received MNT had a mean decrease of HbA1c of 0.59% from baseline at 0 and at 3 months [17].

We found that FPG was 215 mg/dl with an average of 150-492 mg/dl in the pre-intervention, while the average FPG was 150 mg/dl with an average of 110-280 mg/dl and a significant mean difference was 73.97 ± 51.1 mg/dl in the post-intervention. This agrees with a study in Iran that showed an FPG decrease of 21 ± 55 mg/dl in the intervention group (P = 0.028) [16], as well as another study in Iran that reported a significant reduction in FPG (8.85 mg/dl) [7]. This is concomitant with an RCT that revealed a mean decrease of FPG 33.4 mg/dL after 3 months [17]. This contrasts the findings of a study conducted in the USA which reported no change in FPG [15]. This discrepancy could be explained by good patients' adherence to a diabetic diet after attending the educational program in the current study. Other laboratory changes in the lipid profile found in the current study showed a statistically significant decrease as mean differences in the total cholesterol, HDL cholesterol, LDL cholesterol, and triglyceride were 26.03 ± 37.13, 1.83 ± 4.11, 28.54 ± 32.71, 28.90 ± 59.97, respectively. These findings are similar to two studies conducted in India and the USA that revealed a significant reduction in total cholesterol (19.6 and 4.06 mg/dl, respectively) [7,17]. This is in contrast to the findings of a study conducted in the USA which reported no difference in the total serum cholesterol, and LDL. Likewise, this could be due to a different culture and dietary habits between our sample and the previous one, which depended on junk food and fatty foods [15].

**Limitations:** the emerging COVID-19 pandemic crisis, with its social and physical restrictions, led the investigator to provide follow-up visits by phone and communicate using social networking media, e.g., WhatsApp messages. Furthermore, our results cannot be generalized given our interventional study's sample size of T2DM patients.

## Conclusion

This study provided evidence to support the incorporation of MNT as a fundamental component of T2DM management. FPG, HbA1c, weight, BMI, cholesterol, and SBP all improved due to treatment. **Recommendations:** T2DM patients might be helped by making MNT a regular part of their care.

### What is known about this topic

- *All providers should refer patients with T2DM for personalized MNT as the foundation of their care;*
- *MNT delivered by a registered dietician/registered dietician nurse (RD/RDN) is associated with an HbA1c absolute decrease of 0.3-2.0% for patients with T2DM;*
- *In people with insulin resistance, modest weight loss is advantageous, but medicines must usually be paired with MNT when the disease develops into insulin insufficiency*.

### What this study adds

- *MNT is effective in glycemic control of T2DM, tailored to every patient while considering affordability according to socioeconomic status;*
- *MNT does not control HbA1c only, but it also causes a decrease in weight, lipid profile, and BP; hence all the metabolic profiles will be controlled without adding more drugs and increasing insulin sensitivity;*
- *The “plate method” is the most straightforward approach in meal planning compared to other methods*.
